# The conserved transcription factors, MYB115 and MYB118, control expression of the newly evolved benzoyloxy glucosinolate pathway in *Arabidopsis thaliana*

**DOI:** 10.3389/fpls.2015.00343

**Published:** 2015-05-13

**Authors:** Yuanyuan Zhang, Baohua Li, Dongxin Huai, Yongming Zhou, Daniel J. Kliebenstein

**Affiliations:** ^1^National Key Laboratory of Crop Genetic Improvement, College of Plant Science and Technology, Huazhong Agricultural UniversityWuhan, China; ^2^Department of Plant Sciences, University of California, DavisDavis, CA, USA; ^3^DynaMo Center of Excellence, Copenhagen Plant Science Centre, University of CopenhagenCopenhagen, Denmark

**Keywords:** neo-functionalization, sub-functionalization, glucosinolates, co-expression, R2R3-MYB

## Abstract

The evolution of plant metabolic diversity is largely driven by gene duplication and ensuing sub-functionalization and/or neo-functionalization to generate new enzymatic activities. However, it is not clear whether the transcription factors (TFs) regulating these new enzyme encoding genes were required to co-evolve with these genes in a similar fashion or if these new genes can be captured by existing conserved TFs to provide the appropriate expression pattern. In this study, we found two conserved TFs, MYB115, and MYB118, co-expressed with the key enzyme encoding genes in the newly evolved benzoyloxy glucosinolate (GLS) pathway. These TFs interacted with the promoters of the GLS biosynthetic genes and negatively influenced their expression. Similarly, the GLS profiles of these two TFs knockouts showed that they influenced the aliphatic GLS accumulation within seed, leaf and flower, while they mainly expressed in seeds. Further studies indicated that they are functionally redundant and epistatically interact to control the transcription of GLS genes. Complementation study confirmed their roles in regulating the aliphatic GLS biosynthesis. These results suggest that the newly evolved enzyme encoding genes for novel metabolites can be regulated by conserved TFs, which helps to improve our model for newly evolved genes regulation.

## Introduction

Plants need to address and survive ever changing biotic and abiotic environmental stresses without the capacity to move to avoid these stresses. One mechanism by which plants cope with these stresses is through the production of defensive secondary metabolites. The diversity of stresses has led to an equal diversification of the secondary metabolites with a huge number of lineage and species specific compounds (Wink, 1988). A key mechanism hypothesized to generate this diversity in plant metabolism is gene duplications. Most eukaryotic genomes have undergone whole-genome duplications, especially angiosperms (Wolfe and Shields, 1997; Vision et al., 2000; Blanc et al., 2003). Over time, these duplicated genes might be lost or silenced, maintain the ancestral function, or undergo functional divergence either through neo- or sub-functionalization (Lynch and Conery, 2000). Recent work is beginning to highlight the role of gene duplication in amplifying the biosynthetic capacity of plants but this equally requires an increase in transcriptional control over these new genes (Kliebenstein and Osbourn, 2012; Kliebenstein, 2013).

Over the past two decades, glucosinolates (GLSs) present almost exclusive in the order Brassicales including model plant *Arabidopsis thaliana* have evolved as a model system for the study of secondary metabolite diversity in plants (Sønderby et al., 2010b). GLS are sulfur-rich, nitrogen-containing, amino acid-derived compounds (Agerbirk and Olsen, 2012) involved in aiding the plant to resist a myriad of attacking herbivores and pathogens and controlling the plants fitness within the field (Kliebenstein et al., 2002; Bednarek et al., 2009; Clay et al., 2009; Fan et al., 2011; Stotz et al., 2011). There have been more than 200 different GLSs identified in plants (Clarke, 2010), with *A. thaliana* containing at least 40 GLSs. The GLS can generally be classified into three groups according to their amino acid precursors: aliphatic—methionine, valine, isoleucine or leucine, benzolic—phenylalanine or tyrosine and indolic—tryptophan GLS (Fahey et al., 2001; Kliebenstein et al., 2001c). The biosynthesis of aliphatic GLS can be divided into three steps: side-chain elongation of precursor amino acid, core structure pathway, and side-chain modifications. The side-chain modifications of aliphatic GLS greatly contributes to structural and functional diversity of GLS and appear to be the newest evolved genes within the pathway, indicating that these steps are a good model for how newly evolved biosynthetic processes may be integrated into a regulatory system (Sønderby et al., 2010b).

The near complete identification of enzymes in *A. thaliana* GLS biosynthesis has provided support for the role of gene duplication and neo- and/or sub-functionalization across multiple loci to provide the basis of the diversity in secondary metabolites (Hansen et al., 2001, 2007; Kliebenstein et al., 2001a; Chen et al., 2003; Textor et al., 2007; Kliebenstein, 2008; Li et al., 2008). For example, side-chain length variation of methionine-derived aliphatic GLS is controlled by differential expression of three tandem duplicate genes, *MAM1*, *MAM2*, and *MAM3* (Kliebenstein et al., 2001a; Kroymann et al., 2001, 2003). *MAM1* is capable of doing two elongation cycles while *MAM2* controls a single cycle and *MAM3* can catalyze through 6 elongation cycles (Kroymann et al., 2001, 2003; Field et al., 2004; Textor et al., 2007).

Similarly, neo-functionalization of two tandem 2-oxoglutarate-dependent dioxygenases: *AOP2* and *AOP3*, catalyze differential conversion of methylsulfinylalkyl-GLSs (MS-GLSs) to either the alkenyl GLS (AOP2) or chain-length specific hydroxyalkyl GLS (AOP3) (Kliebenstein et al., 2001c). A role for both tandem and whole-genome duplication in diversifying the pathway comes from five flavin-monooxygenases (*FMO_S_*) (*FMO_GS−OX1−5_*) which convert methylthioalkyl-GLSs (MT-GLSs) to methylsulfinylalkyl-GLSs (MS-GLSs). These genes differentially convert MT-GLSs to MS-GLSs and they arose from a whole-genome duplication followed by ensuing tandem duplication at each locus and sub-functionalization (Hansen et al., 2007; Li et al., 2008). Similar evidence is also emerging from analysis of the indolic GLS pathway (Hull et al., 2000; Mikkelsen et al., 2000; Bednarek et al., 2009; Clay et al., 2009; Pfalz et al., 2009, 2011).

An understudied aspect of the evolution of new biosynthetic pathways is how their accompanying regulatory machinery arises. Are they controlled by transcription factors (TFs) that are equally derived from gene duplication or are these new pathways controlled by conserved TFs (Kliebenstein and Osbourn, 2012; Kliebenstein, 2013)? Recent observations in the GLS regulatory machinery have begun to provide evidence for both options. Two main TF families have been linked to regulating GSL production, the myeloblastosis (MYB) and myelocytomatosis (MYC) TFs (Dubos et al., 2010; Schweizer et al., 2013; Frerigmann et al., 2014; Li et al., 2014). The MYBs involve a paralogous group of genes belonging to subgroup 12 of the R2R3 MYB family, which are proved to be functionally redundant and specific involved in the transcriptional regulation of aliphatic GLSs (MYB28 family: *MYB28*, *MYB29*, and *MYB76*) (Gigolashvili et al., 2007, 2008, 2009; Hirai et al., 2007; Sønderby et al., 2007, 2010a) and indolic GLSs (MYB34 family: *MYB34*, *MYB51*, and *MYB122*) (Malitsky et al., 2008; Gigolashvili et al., 2009; Frerigmann and Gigolashvili, 2014). These MYBs are unique to the GLS containing Brassicales and arose via whole-genome and tandem duplication with ensuing neo-functionalization to focus on one branch of the GLS pathway (Bekaert et al., 2012). In contrast to MYB TFs, the MYC family shows evidence wherein conserved jasmonate signaling TFs have captured the regulation of the newly evolved GLS pathway (Dombrecht et al., 2007; Fernández-Calvo et al., 2011; Schweizer et al., 2013; Frerigmann et al., 2014; Li et al., 2014). The MYCs are encoded by four genes that arose via gene duplication and there is evidence of sub-functionalization among them (Heim et al., 2003; Fernández-Calvo et al., 2011; Niu and Figueroa, 2011; Schweizer et al., 2013; Frerigmann et al., 2014). More recently a broad set of new conserved and evolutionarily limited TFs were added to the list of potential regulators for the GLS pathway (Li et al., 2014). Thus, there is evidence for TFs controlling the GLS pathway to co-evolve with the pathway (MYB_S_) or to have captured the pathway (MYCs). This suggests that more work is needed to figure out if one or the other model is more prevalent.

**Figure 1:**
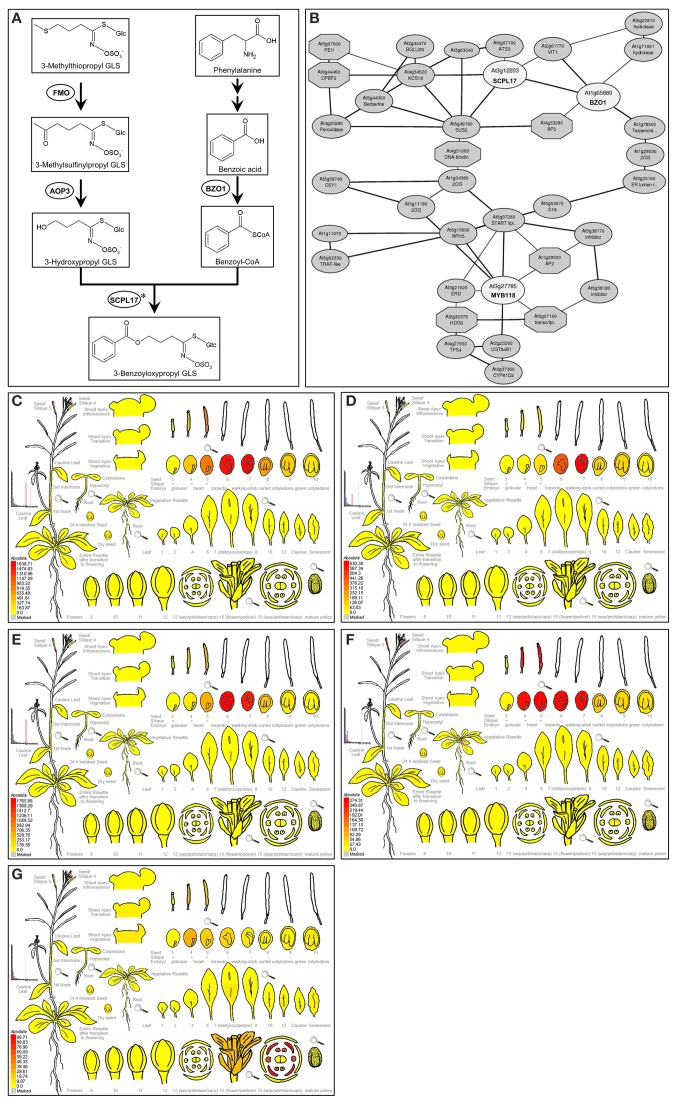
**Expression pattern of *AOP3*, *BZO1*, *SCPL17*, and *MYB118*. (A)** Proposed biosynthetic pathway leading to 3-benzoyloxypropyl GLS in *A. thaliana*. The proposed biosynthesis of 4-benzoyloxybutyl GLS proceeds in a similar manner, but with a 4-carbon aliphatic GLSs precursor. Metabolites are shown in boxes and enzymes are shown in circles. Arrows between compounds represent the number of putative enzymatic reactions. Asterisks partially characterized enzyme. **(B)** The co-expression network analysis of *BZO1*, *SCPL17*, and *MYB118* using the ATTED-II database (Obayashi et al., 2014). The TFs and genes are shown in circles. **(C–G)** e-FP display of transcript accumulation patterns across a variety of Arabidopsis organs. Arabidopsis e-FP browser (Winter et al., 2007) presents the transcript accumulation pattern of *AOP3* **(C)**, *BZO1* **(D)**, *SCPL17* **(E)**, *MYB118* **(F)**, and *MYB115* **(G)** in the siliques. In all cases, red indicates higher levels of transcript accumulation and yellow indicates a lower level of transcript accumulation.

To generate more evidence assessing which of these two models may be more likely, we focused on the transcriptional regulation of a branch of the GLS pathway that recently evolved within the *A. thaliana* lineage (Kliebenstein and Osbourn, 2012; Kliebenstein, 2013). Benzoyloxy GLS (BZ-GLS) are found in *A. thaliana* but not in its close relatives, suggesting that this pathway arose within the *A. thaliana* lineage is very young providing an opportunity to investigate how very young biochemical pathways are regulated. The production of BZ-GLS within the Col-0 accessions of *A. thaliana* requires the involvement of side-chain modifying enzymes encoded by the *AOP3*, *BZO1*, and *SCPL17* genes (Figure 1A). This pathway is transcriptionally limited to the developing seed suggesting that it is precisely regulated at the transcriptional level (Kliebenstein et al., 2001c, 2007; Lee et al., 2012). To test if this new pathway is regulated by a novel lineage specific TF or captured by an existing conserved pathway, we searched for candidate TFs regulating BZ-GLS-related genes, *AOP3, BZO1*, and *SCPL17* using a comprehensive strategy based on transcription co-expression analysis (Obayashi et al., 2014). This identified the homologous TFs *MYB115* and *MYB118* as likely candidates for controlling the transcription of this pathway (Dubos et al., 2010). These genes had previously been characterized as conserved TFs that regulate maturation-related genes and promote the vegetative-to-embryonic transition (Wang et al., 2009; Zhang et al., 2009; Barthole et al., 2014). They had not previously been associated with the regulation of GLS biosynthesis. Here, we functionally validate that the two R2R3-MYB TFs from *A. thaliana*, *MYB115* and *MYB118*, play important roles in transcriptional regulation of aliphatic GLS biosynthetic pathway for the production of the new BZ-GLS pathway. We further investigate the epispastic interactions between *MYB115*/*MYB118* and some key genes in GLS biosynthetic pathway, in order to describe the regulation model of *MYB115* and *MYB118* in aliphatic GLS biosynthesis. This shows that the newly evolved BZ-GLS pathway is regulated by conserved TFs that previously existed to provide the specific developmental context observed for BZ-GLSs.

## Results

### MYB115 and MYB118 are candidate regulators of GLS biosynthesis using co-expression analysis and yeast one-hybrid assay

Using a comprehensive strategy of co-expression analysis, combined with the transcriptome co-expression profiles from ATTED-II database (Obayashi et al., 2014) and the expression pattern using Arabidopsis e-FP browser (Winter et al., 2007), a total of 300 TFs were shown to highly co-express with BZ-GLS-related genes, *AOP3*, *BZO1*, and *SCPL17*, which are the key genes involved in the side-chain modification pathway of GLS biosynthesis, especially BZ-GLS biosynthesis (Figure 1A). Focusing on R2R3-MYB TFs which are frequently key secondary metabolite regulatory TFs leads to the identification of three R2R3-MYB TFs as candidates, *MYB56*, *MYB58*, and *MYB118* (Figure 1B). From this, only *MYB118* exhibited the same seed-specific expression pattern as that of *AOP3*, *BZO1*, and *SCPL17* (Figures 1C–F). MYB118 (AT3G27785) belongs to subgroup 25 of the R2R3-MYB TF family containing six members (Dubos et al., 2010). *MYB115* (At5g40360) is the most closely related to *MYB118* in the same subgroup, and overexpression of the two TFs showed similar phenotypes suggesting that they have similar functions (Wang et al., 2009; Zhang et al., 2009; Barthole et al., 2014). Thus, we hypothesized that *MYB118* and *MYB115* may both transcriptionally regulate the BZ-GLS pathway.

**Figure 2:**
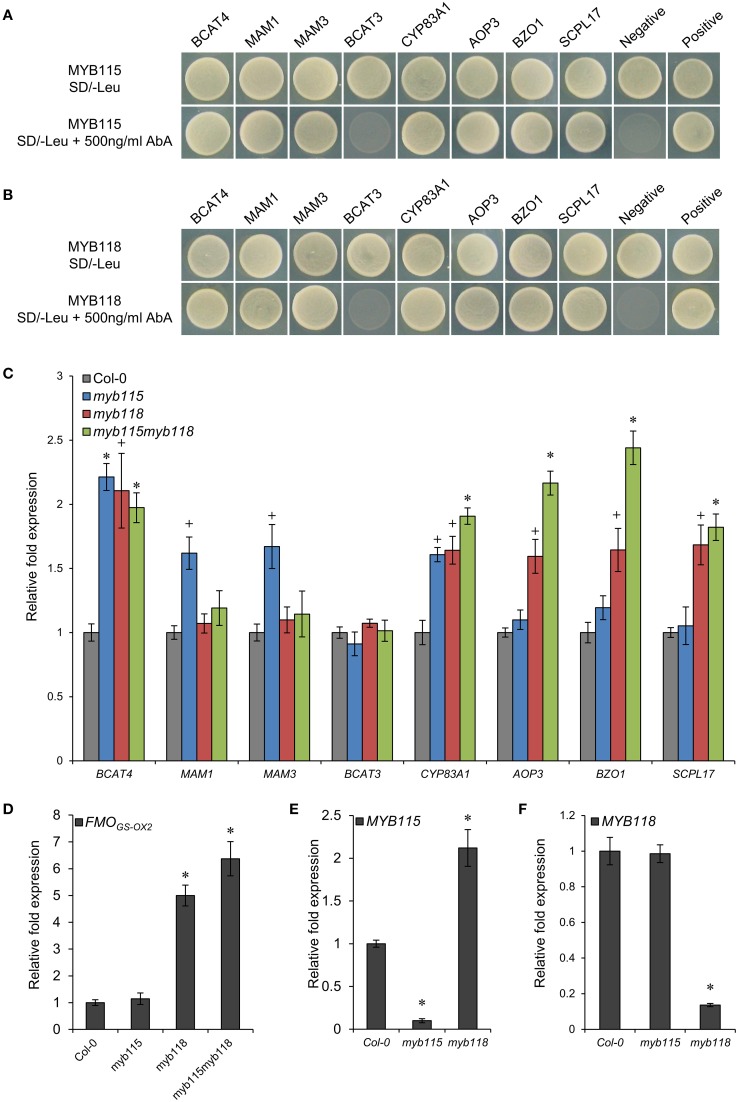
**Identification of interactions between MYB115/MYB118 and aliphatic GLS biosynthesis-related genes. (A,B)** Yeast one-hybrid assays showing binding of MYB115 **(A)** and MYB118 **(B)** to the promoters of aliphatic GLS biosynthesis-related genes. The Y1H Gold yeast strains growth in the absence (top) and presence of 500 ng ml^−1^ Aureobasidin A (AbA) on the SD-Leu plates is shown. The mutant pABAi vector was used as the negative control and the p53 vector was used as the positive control. **(C–F)** qRT-PCR analysis of aliphatic GLS biosynthesis-related genes measured in the seeds of Col-0, *myb115*, *myb118*, and *myb115myb118* knockouts. The relative expression values of genes were measured on developing seeds harvested 6 and 7 days after pollination. Ubiquitin-conjugating enzyme 21 (*UBC21*) gene expression level was used as a constitutive control. Values are the means and SE of three independent mRNA extractions. The significant differences is shown as ^+^(*P* < 0.05) or ^*^ (*P* < 0.01) using *t*-tests.

To validate the potential regulatory roles of *MYB115* and *MYB118* in the aliphatic GLS biosynthetic pathway, a yeast one-hybrid study (Y1H) was used to test their interactions with eight promoters of aliphatic GLS pathway genes which were mainly involved in the side-chain elongation and modification of GLS biosynthesis (Figure 2). Surprisingly, the result showed that *MYB115* and *MYB118* not only bound the promoters of BZ-GLS modification genes (*BZO1*, *SCPL17*, and *AOP3*) but also bound the promoters of three side-chain elongation genes (*BCAT4*, *MAM1*, and *MAM3*) and a core structure pathway gene (*CYP83A1*). Thus, the Y1H data show that *MYB118* and *MYB115* can bind a large number of promoters in the pathway and might have broader impact on the regulation of GLS biosynthesis than simply the BZ-GLS.

To validate if these Y1H predicted promoter interactions may alter the transcription of the downstream genes in planta, we analyzed the expression patterns of these genes by qRT-PCR in *MYB115* and *MYB118* gene-knockout plants, designated as *myb115* and *myb118*, respectively, and double *myb115myb118* mutants (Figures S1A,B). Total RNA was isolated from the seeds harvested 6 and 7 days after pollination from three independent mRNA extractions. The result showed that the expression levels of the genes involved in side-chain modification of GLS biosynthesis [*AOP3*, *BZO1*, *SCPL17*, and *FMO_GS−OX2_* which co-expressed with *BZO1*, *SCPL17*, and *MYB118* in the siliques (Figure S2A), but it also have some expression in vegetative tissue (Figure S2B)] were significantly increased in *myb118* while in *myb115* they were similar to the wild-type Col-0 controls (WT) (Figures 2C,D). In addition, the double mutant *myb115myb118* lead to a further increase in expression of these genes above either single mutant genotype. In contrast to the side-chain modification genes, the chain elongation genes showed different expression patterns in single and double mutants. The expression levels of *BCAT4* and CYP83A1 were significantly and equally increased in all *MYB115* and *MYB118* single and double knockouts in comparison with wild-type Col-0. In contrast to *BCAT4*, BCAT3 had no altered expression in any genotype which agrees with its promoter showing no evidence of interaction with either *MYB115* or *MYB118*. For the two genes that centrally regulate side-chain elongation, *MAM1* and *MAM3*, only *myb115* showed a significant change in expression levels in comparison with wild-type Col-0. This increased expression was abolished in the double mutant. Taken together, MYB118 and MYB115 can regulate GLS biosynthesis in the seeds of Arabidopsis and function to genetically repress the expression of their target genes.

### Multiple GLS phenotypes in *MYB115* and *MYB118* single mutant seeds

**Figure 3:**
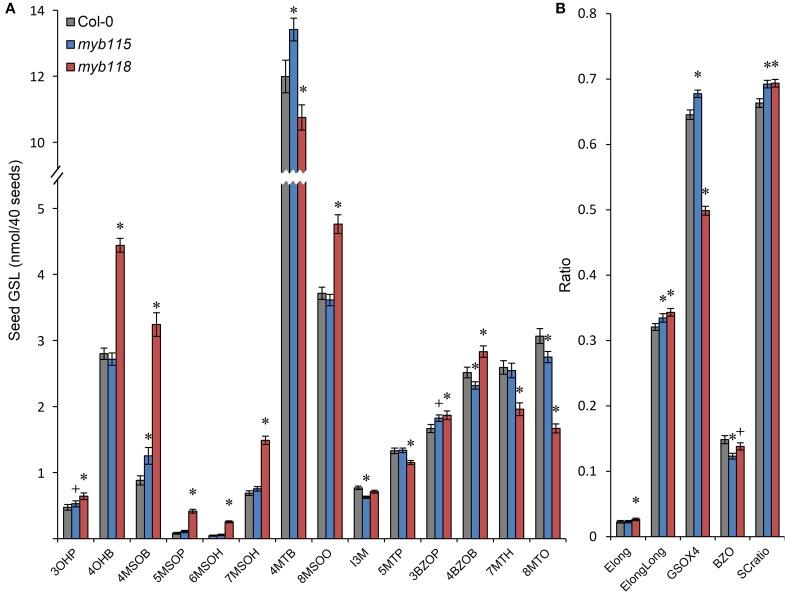
**GLS content in seeds of *myb115* and *myb118* knockout mutants. (A)** GLS contents per 40 seeds was measured by HPLC (Means and SE, *n* = 48). The data are obtained from six independent experiments and were analyzed via ANOVA. A statistically significant change in *myb115* and *myb118* knockout mutants compared with wild type is shown as ^*^(*P* < 0.01) or ^+^(*P* < 0.05) on the bar. (For GLS abbreviations, see Table 1). **(B)** Statistical analysis of biosynthetic ratios of GLS data in **(A)**. Elong = 3C/(3C+4C) ElongLong = 7C/(7C+8C) GSOX4 = 4MTB/(4MTB+4MSOB+4OHB+4BZOB) BZO = 4BZOB/(4MTB+4MSOB+4OHB+4BZOB) SCratio = (3C+4C)/Total Aliphatic.

To test if the predicted transcriptional regulatory function of *MYB115* and *MYB118* leads to altered GLS accumulation, we analyzed GLS accumulation in gene-knockout plants (*myb115* and *myb118*), both of which grew normally (for GLS abbreviations, see Table 1 and Table S1). Because the over-expression lines show extremely stunted growth and sterility we were unable to measure GLS phenotypes in these lines (Wang et al., 2009; Zhang et al., 2009; Barthole et al., 2014). In *myb118* seeds (Figure 3; Table S2), the contents of MS-GLSs, OH-GLSs and BZ-GLSs were significantly increased in agreement with the induced expression of the associated genes. In contrast, the content of MT-GLSs was decreased, whereas the content of indolic GLS (I3M) did not change significantly in comparison with WT. Notably, the phenotypic changes on GLS content in *myb118* seeds are comparable, with opposite sign to the ones of *mby28* and *myb29* single mutants (Hirai et al., 2007; Sønderby et al., 2007), indicating that MYB118 may have an equally important impact on the transcriptional network on controlling GLS production *in planta*.

**Table 1:** **Glucosinolate (GLS) abbreviations in this study**.

**Abbreviation**	**GLS type**	**GLS name**
3MTP	Aliphatic 3C	3-methylthiopropyl GLS
3MSOP	Aliphatic 3C	3-methylsulfinylpropyl GLS
3OHP	Aliphatic 3C	3-hydroxypropyl GLS
3BZOP	Aliphatic 3C	3-benzoyloxypropyl GLS
4MTB	Aliphatic 4C	4-methylthiobutyl GLS
4MSOB	Aliphatic 4C	4-methylsulfinylbutyl GLS
4OHB	Aliphatic 4C	4-hydroxybutyl GLS
4BZOB	Aliphatic 4C	4-benzoyloxybutyl GLS
2OH-Butenyl	Aliphatic 4C	2-hydroxy-but-3-enyl GLS
5MSOP	Aliphatic 5C	5-methylsulfinylpentyl GLS
6MTH	Aliphatic 6C	6-methylthiohexyl GLS
6MSOH	Aliphatic 6C	6-methylsulfinylhexyl GLS
7MTH	Aliphatic 7C	7-methylthioheptyl GLS
7MSOH	Aliphatic 7C	7-methylsulfinylheptyl GLS
8MTO	Aliphatic 8C	8-methylthiooctyl GLS
8MSOO	Aliphatic 8C	8-methylsulfinyloctyl GLS
4OH-I3M	Indolic	4-hydroxy-indol-3-ylmethyl GLS
I3M	Indolic	indol-3-ylmethyl GLS
4MO-I3M	Indolic	4-methoxy-indol-3-ylmethyl GLS
NMO-I3M	Indolic	N-methoxy-indol-3-ylmethyl GLS

Abbreviations, type, and name of glucosinolates in this study.

In agreement with the absence of a significant transcriptional change, the phenotypic changes of GLS content in *myb115* mutants are much weaker than the ones of myb118. 3OHP, 4MSOB, 4MTB, and 3BZOP GLSs are statistically significantly but only slightly increased, while the levels of 4BZOB and 8MTO GLSs were similarly reduced in a significant but subtle manner (Figure 3; Table S2). These data indicated that *MYB115* might also control the aliphatic GLS biosynthetic genes in seed. Thus, the GLS changed in *myb115* or *myb118* in comparison with the wild type, such as MS-GLSs, OH-GLSs and BZ-GLSs, were related to the function of *AOP3* or *BZO1* in GLS biosynthesis. These findings support the result of co-expression analysis that *MYB115* and *MYB118* regulate the *AOP3*/*BZO1* related GLS phenotypes. Notably, both *MYB118* and *MYB115* negatively influence the regulation of most GLS accumulation in agreement with the observed transcriptional change. This is in contrast to the previous identified MYB transcriptional regulators including *MYB28* and *MYB29* which are all positive regulators for the pathway (Gigolashvili et al., 2007; Hirai et al., 2007; Sønderby et al., 2007).

### *MYB115* and *MYB118* also affect the content of GLS in leaf and flower organs

**Figure 4:**
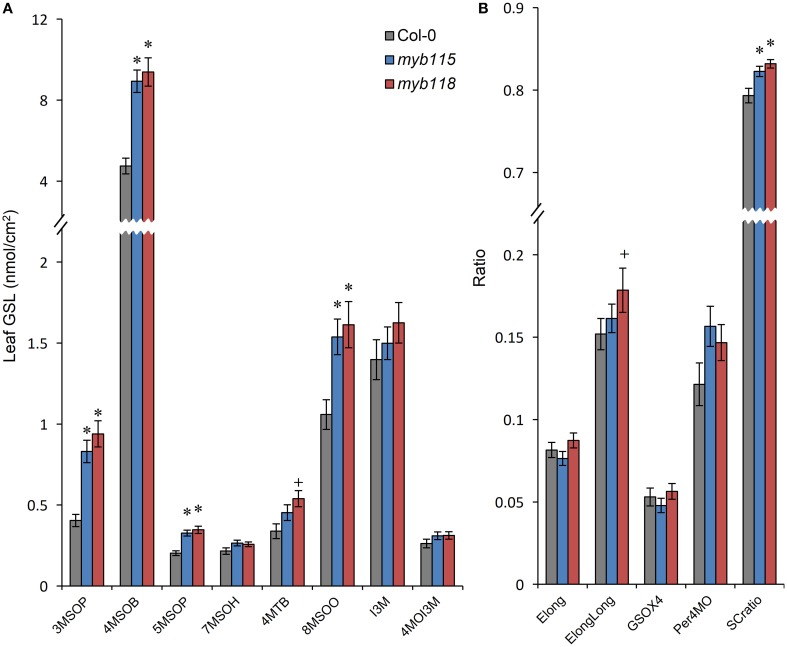
**GLS content in leaves of *myb115* and *myb118* knockout mutants. (A)** GLS accumulation in 4-week-old rosette leaves. (Means and SE, *n* = 48). The data are sums of six independent experiments and were analyzed via ANOVA. A statistically significant change in *myb115* and *myb118* knockout mutants compared with wild type is shown as ^*^(*P* < 0.01) or ^+^(*P* < 0.05) on the bar. **(B)** Statistical analysis of biosynthetic ratios of GLS data in **(A)**.

To test if *MYB115* and *MYB118* can influence GLS biosynthesis in additional tissues, we measured GLS accumulation in leaves and flowers of the single mutants. While both *MYB118* and *MYB115* are considered to be expressed specifically to reproductive tissues (Wang et al., 2009; Zhang et al., 2009; Barthole et al., 2014), they do have low residual expression in vegetative tissue (Figures 1F,G). Interestingly, both single mutants lead to increased aliphatic GLS accumulation in vegetative tissues with no corresponding change in indolic GLSs (Figure 4; Table S2). In contrast to the seeds, *myb115* showed similar GLS phenotypes to *myb118 in* leaves. These data suggest that *MYB118* and *MYB115* can alter the accumulation of aliphatic GLS within the leaf even with low residual transcript accumulation.

**Figure 5:**
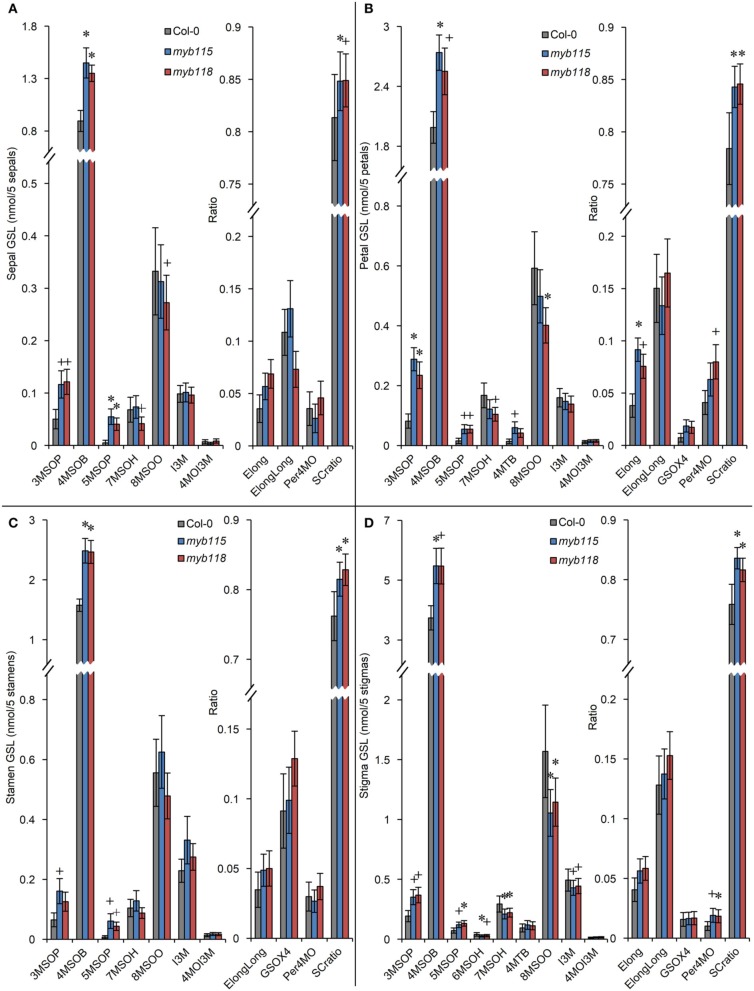
**GLS content in flower organs (sepal, petal, stamen, stigma) of *myb115* and *myb118* knockout mutants**. GLS contents of sepals **(A)**, petals **(B)**, stamens **(C)**, and stigmas **(D)** of 5 flowers and the respective statistical analysis of biosynthetic ratios. (Means and SE, *n* = 24). The data are combined from four independent experiments and analyzed via ANOVA. A statistically significant change in *myb115* and *myb118* knockout mutants compared with wild type is shown as ^*^(*P* < 0.01) or ^+^(*P* < 0.05) on the bar.

Transcript data showed that *MYB115* shows a distinct expression pattern within the flower where it is localized to petals and stamens, but not in sepals and carpels (Figure 1G). To investigate the effect of this specific express pattern, we analyzed the GLSs of *myb115* and *myb118* in each flower organs (sepal, petal, stamen, and stigma). This showed that all flower organs of *myb115* and *myb118* mutants had increased levels of short chain MS-GLSs, 3MSOP, 4MSOB, and 5MSOP (Figure 5; Table S2). The only tissue specific change was an alteration of long chain MS-GLSs (7MSOH and 8MSOO) that was largely limited to the petal and stigmas of the single mutants. Thus, the *MYB118* and *MYB115* TFs influence GLS accumulation throughout the plant.

### Complementation study confirmed the phenotypes of *MYB115* and *MYB118* single mutants

**Figure 6:**
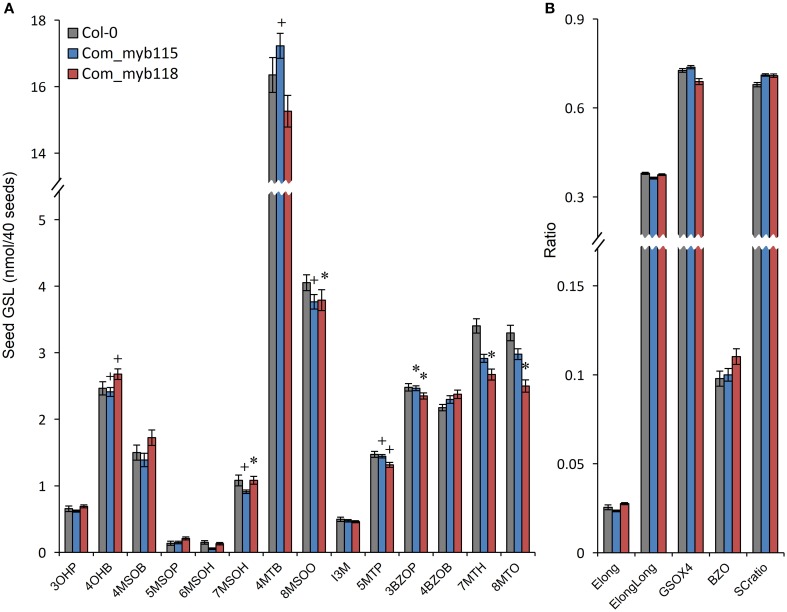
**GLS content in seeds of *myb115* and *myb118* complementation lines**. Nested ANOVAs were utilized to test for significant differences in the GLS content of three independent *myb115* or *myb118* complementation lines introduced into the *myb115* or *myb118* single mutant backgrounds to test whether the defects of single mutant can be complemented. **(A)** GLS contents of 40 seeds (Means and SE, *n* = 36). The data are sums of two independent experiments with four replicates per genotype and were analyzed via ANOVA. A statistically significant change in *myb115* and *myb118* complementation lines compared with wild type is shown as ^*^(*P* < 0.01) or + (*P* < 0.05) on the bar. **(B)** Statistical analysis of biosynthetic ratios of GLS data in **(A)**.

To confirm the metabolite phenotypes found in the *myb115* and *myb118* mutant, we transformed the respective T-DNA lines mutants with the genomic region of the each MYBs respectively. We then tested the seed GLS content of three independent *myb115* and *myb118* complementation lines by nested ANOVA wherein we tested for both a difference from the WT control and between the complementation lines. No significant differences were found amongst the complementation lines and we thus focused on the genotypic differences. This showed that introducing the WT *MYB115* gene into the *myb115* mutant abolished the observed increase in short-chain GLSs and returned the genotype to a WT phenotype (Compare Figure 6 to Figure 3; Tables S2, S3). Similarly, introducing the WT *MYB118* gene into the *myb118* mutant complemented the altered short-chain GSL accumulation phenotype. Thus, the short-chain GLS phenotypes in the two *myb115* and *myb118* TDNA insertion lines were complemented by the DNA fragment harboring the intact *MYB115* and *MYB118* genes (Figure 6; Table S3). The long-chain GLS levels were not fully complemented but were closer to wild-type than the corresponding single mutant suggesting that these had been partially complemented. These results strongly support the roles of *MYB115* and *MYB118* in regulating the aliphatic GLS biosynthesis.

### Double knockouts show an interaction between *MYB115* and *MYB118* in aliphatic GLS biosynthesis

**Figure 7:**
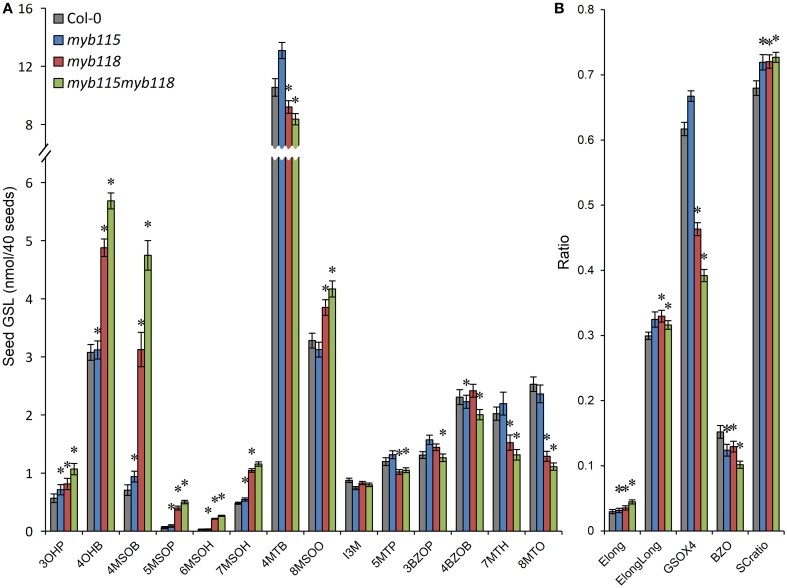
**GLS content in seeds of *myb115*, *myb118*, and *myb115myb118* knockout mutants**. The data are from two independent experiments and were analyzed via ANOVA. Asterisks are placed above a genotype to show if a main effect or interaction term is significant (*P* < 0.05). An asterisk above the *myb115* or *myb118* shows that there is a main effect of that gene within the ANOVA while an asterisk above *myb115myb118* shows that there was a statistically significant double mutant interaction indicating epistasis between *myb115* and *myb118*. **(A)** GLS content of 40 seeds. (Means and SE, *n* = 28). **(B)** Statistical analysis of biosynthetic ratios of GLS data in **(A)**.

The qRT-PCR work had suggested that there may be an epistatic interaction between *MYB115* and *MYB118* in controlling aliphatic GLS accumulation (Figure 2C). To test if *MYB115* and *MYB118* may epistatically control GLS accumulation, the double mutant was grown under controlled conditions, together with wild-type control and *myb115* and *myb118* single mutants, and the content of GLS in seeds was measured. The GLS levels were then analyzed using a Two-Way ANOVA to parse out the effects of the single mutants and any interaction effects. There was a statistically significant effect of the *myb115myb118* double mutant on the short-chain GLS where the double mutant showed a higher accumulation of the OH-GLSs and MS-GLSs greater than expected from the single mutant effects (Figure 7; Table S4). Interestingly, this did not correspond to a significant double mutant increase in BZ-GLS as was expected based on the qRT-PCR (Figure 2C). This suggests that the benzoyl moiety may be limiting in these genotypes. In contrast to the GLS level in seeds, *myb115myb*118 didn't show any evidence of epistatic interaction when measuring leaf GLS accumulation (Figure S3; Table S4). In agreement with the observed epistasis between *MYB115* and *MYB118*, the expression level of *MYB115* was significantly increased in *myb118* knockouts in comparison with wild-type Col-0. In contrast, *MYB118* expression in the *myb115* genotype was similar to the wild-type Col-0 (Figures 2E,F). This result suggests that in the *myb118* knockout, the function of *MYB118* is partly replaced by elevated function of *MYB115* suggesting that MYB118 regulates MYB115. Thus, *MYB115* and *MYB118* epistatically interact to control the transcription of GLS genes and GLS accumulation in Arabidopsis seeds (Figures 2, 7).

### *MYB115* and *MYB118* show different interactions with the genes involved in side-chain modification pathway of GLS biosynthesis

**Figure 8:**
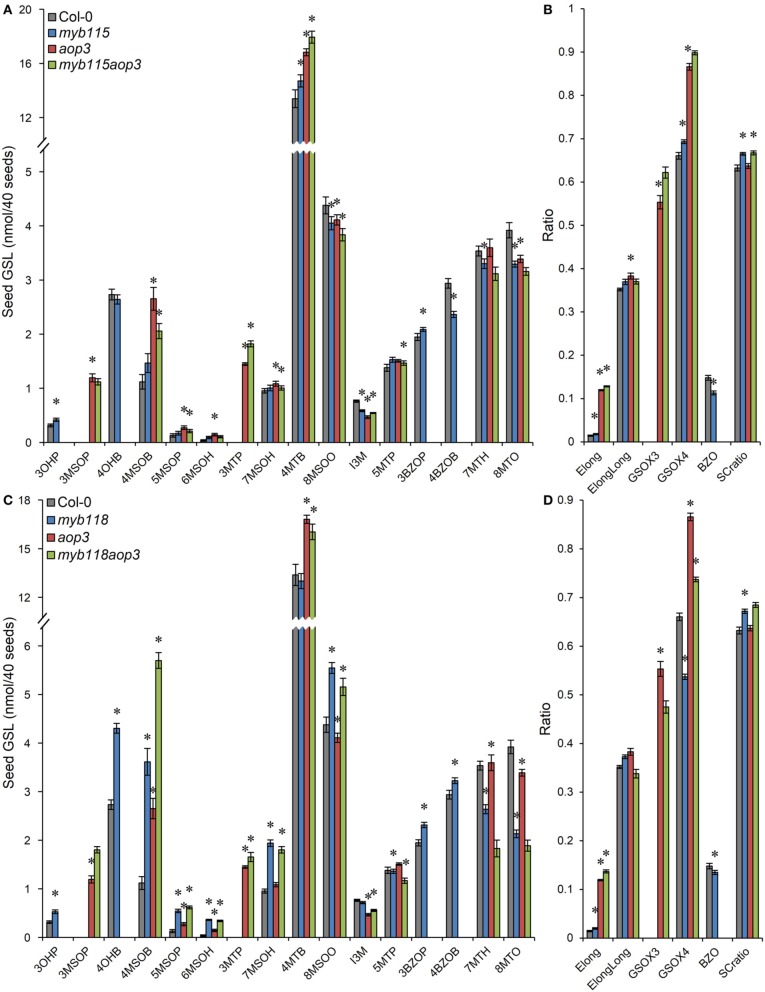
**GLS content in seeds of *myb115aop3* and *myb118aop3* double knockouts. (A)** GLS accumulation in 40 seeds of wild-type Col-0, *myb115*, *aop3*, and *myb115aop3* knockouts (Means and SE, *n* = 16). The data are combined from two independent experiments and were analyzed via ANOVA. Asterisks are placed above a genotype to show if a main effect or interaction term is significant (*P* < 0.05). An asterisk above the *myb115* or *aop3* shows that there is a main effect of that gene within the ANOVA while an asterisk above *myb115aop3* shows that there was a statistically significant double mutant interaction indicating epistasis between *myb115* and *aop3*. **(B)** Statistical analysis of biosynthetic ratios of GLS data in **(A)**. **(C)** GLS accumulation in 40 seeds of wild-type Col-0, *myb118*, *aop3*, and *myb118aop3* knockouts (Means and SE, *n* = 16). The data are the combined results from two independent experiments and were analyzed via ANOVA. Asterisks are placed above a genotype to show if a main effect or interaction term is significant (*P* < 0.05). An asterisk above the *myb118* or *aop3* shows that there is a main effect of that gene within the ANOVA while an asterisk above *myb118aop3* shows that there was a statistically significant double mutant interaction indicating epistasis between *myb118* and *aop3*. **(D)** Statistical analysis of biosynthetic ratios of GLS data in **(C)**.

**Figure 9:**
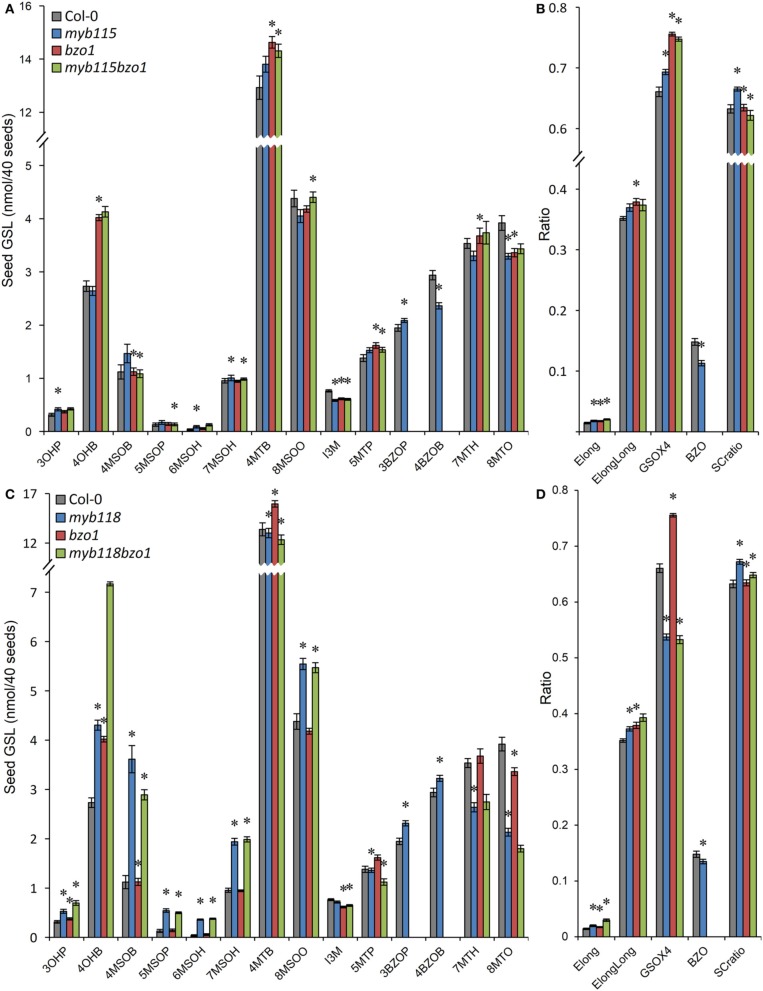
**GLS content in seeds of *myb115bzo1* and *myb118bzo1* double knockouts. (A)** GLS accumulation in 40 seeds of wild-type Col-0, *myb115*, *bzo1*, and *myb115bzo1* knockouts (Means and SE, *n* = 16). The data are combined from two independent experiments and were analyzed via ANOVA. Asterisks are placed above a genotype to show if a main effect or interaction term is significant (*P* < 0.05). An asterisk above the *myb115* or *bzo1* shows that there is a main effect of that gene within the ANOVA while an asterisk above *myb115bzo1* shows that there was a statistically significant double mutant interaction indicating epistasis between *myb115* and *bzo1*. **(B)** Statistical analysis of biosynthetic ratios of GLS data in **(A)**. **(C)** GLS accumulation in 40 seeds of wild-type Col-0, *myb118*, *bzo1*, and *myb118bzo1* knockouts (Means and SE, *n* = 16). The data are combined from two independent experiments and were analyzed via ANOVA. Asterisks are placed above a genotype to show if a main effect or interaction term is significant (*P* < 0.05). An asterisk above the *myb118* or *bzo1* shows that there is a main effect of that gene within the ANOVA while an asterisk above *myb118bzo1* shows that there was a statistically significant double mutant interaction indicating epistasis between *myb118* and *bzo1*. **(D)** Statistical analysis of biosynthetic ratios of GLS data in **(C)**.

We next proceeded to test how the TF mutants interact with mutants in the AOP3 and BZO1 biosynthetic loci to test how the pathway may be diverted in case of a simultaneous regulatory and biosynthetic defect (Kliebenstein et al., 2001c, 2007). To accomplish this, we generated four double mutants, *myb115aop3*, *myb118aop3*, *myb115bzo1*, and *myb118bzo1* double mutants. All the double mutants were grown together with wild-type control and all single mutants (Figure S1), and GLS content of seeds, leaves and flowers were measured and tested via Two-Way ANOVA. In seeds, *MYB115* and *MYB118* both interact with AOP3 but lead to different consequences. A *myb118aop3* double mutant leads to a large accumulation of 4MSOB GLS but no corresponding double mutant increases in 4MTB whereas the *myb115aop3* pushed the GLS to the 4MTB form (Figure 8; Table S5). Similar to the differential AOP3 interactions, the two MYBs had different effects on GSL repartitioning in the *bzo1* background (Figure 9; Table S6). *MYB115* had little to no effect in the bzo1 background with all double mutant phenotypes appearing like the bzo1 single mutant. In contrast, the *myb118bzo1* double mutant had a large increase in the accumulation of the short-chain OH-GSLs at the expense of the MT and MS-GSLs. Thus, *MYB115* and *MYB118* appear to lead to different shifts in GLS partitioning when abolishing parts of the BZ-GSL pathway. Further transcriptomic and metabolomic experiments are needed to better parse these changes.

## Discussion

### MYB115 and MYB118 are conserved TFs that regulate the newly evolved BZO1 and AOP3 produced BZ-GLS in *Arabidopsis thaliana*

Plants can generate a vast diversity of metabolites, which is due to the diversified function of the biosynthetic genes encoding various metabolic enzymes, to cope with environmental changes. The evolution of this diversity is predicted to be driven by gene duplication and consequent neo- and sub-functionalization of enzymes (Kliebenstein, 2008). However, the corresponding pattern of diversification for TFs modulating secondary metabolites is less clear. For example, MYB TFs of subgroup 12 (MYB28/29/76 and MYB34/51/122) have evolved via whole-genome duplication to specifically modulate the GLS pathway genes (Bekaert et al., 2012). In contrast, the conserved JA regulators, MYC TFs of subgroup IIIe (MYC2/3/4/5), also control the GLS pathway but this is likely from regulatory capture rather than specialization for the pathway (Dombrecht et al., 2007; Fernández-Calvo et al., 2011; Schweizer et al., 2013; Frerigmann et al., 2014; Li et al., 2014). Therefore, a question appears: do newly evolved metabolites need regulate by newly evolved TFs? To answer this question, we focused on the regulation of a pathway for a newly evolved GLS, BZ-GLSs, and identified *MYB115* and *MYB118*, two R2R3-MYB TFs co-expressed with BZ-GLS-related genes (*AOP3* and *BZO1*). *MYB115* and *MYB118* epistatically interacted to modulate the expression of the accompanying GLS genes and the respective metabolites in Arabidopsis seeds (Figures 7–9). *MYB115* and *MYB118* are conserved regulators of seed maturation suggesting that the new BZ-GLS biosynthetic pathway was captured by these TFs to impart the specific seed accumulation pattern for the BZ-GLS.

Interestingly, the genetic evidence suggests *MYB115* and *MYB118* function to repress the GLS genes. The identification of a repressor for defense compounds is not unexpected as these compounds are highly patterned in a spatial and ontogenic fashion which requires the combination of activators and repressors to accomplish and to minimize any costs (Brown et al., 2003; Kliebenstein et al., 2007; Wentzell and Kliebenstein, 2008; Wentzell et al., 2008; Züst et al., 2011; Moussaieff et al., 2013). It is also not unusual to identify repressors via Yeast-1-hybrid because of the strength of the activation domain added to the TF can often overcome this activity (Gaudinier et al., 2011; Li et al., 2014; Taylor-Teeples et al., 2014). However, previous work has identified *MYB115* and *MYB118* as activators (Wang et al., 2009; Zhang et al., 2009). There are a number of hypothesis that could explain this discrepancy. First, if a number of TFs have been shown to be activators and repressors depending upon the promoter and other factors binding the promoter (Taylor-Teeples et al., 2014). Second, *MYB115* and *MYB118* may have a passive repressive role where they interfere with the interaction between the activator MYB28/29 and MYCs (Sønderby et al., 2010a; Schweizer et al., 2013; Frerigmann and Gigolashvili, 2014; Frerigmann et al., 2014). Finally, *MYB115* and *MYB118* could be involved in an incoherent feed-forward loop where they activate a unidentified repressor that overcomes any direct activation they may display. The MYB-binding sites have been classified into three types (Romero et al., 1998; Prouse and Campbell, 2012). The MYB118-binding site was shown to belong to type I: pAACnG (where p indicates T or C, and n indicates any nucleotide) (Barthole et al., 2014) with additional influence of the flanking bases (Biedenkapp et al., 1988; Howe and Watson, 1991; Deng et al., 1996). This motif exists in *MYB118* regulated genes that are linked to seed maturation (Barthole et al., 2014). We found, the corresponding element are within the promoters of *AOP3* (TAACAG, at position −1071 to −1066 bp), *BZO1* (TAACAG, at position −345 to −340 bp), *BCAT4* (TAACCG, at position −470 to −465 bp), *CYP83A1* (CGGTTA, at position −132 to −127 bp), *MAM1* (TAACTG, at position −2304 to −2299 bp), MAM3 (TAACTG, at position −1767 to −1762 bp), and *SCPL17* (CTGTTA, at position −6340 to −635 bp), which are all MYB115- and MYB118-binding promoters in Y1H assays (Figures 2A,B). This is similar to the observation that the conserved JA related MYC basic helix–loop–helix (bHLH) TFs also control GLS accumulation via the recruitment of the appropriate promoter elements into the enzyme encoding genes (Chini et al., 2007; Dombrecht et al., 2007; Fernández-Calvo et al., 2011; Schweizer et al., 2013; Frerigmann et al., 2014; Li et al., 2014). This suggests that the promoters obtained elements to allow them to be regulated by these MYBs. Further studies are needed to elucidate the precise molecular function of *MYB115* and *MYB118* in the regulation of GLS biosynthesis, and to find the activators in the seed of BZ-GLS related genes as they are not regulated by the known MYBs or MYCs (Li et al., 2014). One option for these unknown components are the additional genes within the *MYB118* subgroup of R2R3-MYBs which may also play a role in regulating GLS accumulation but this remains to be tested (Dubos et al., 2010). Thus, newly evolved biosynthetic pathways can be rapidly captured by existing TFs to impart highly precise regulatory patterns without requiring the *de novo* evolution of new regulatory TFs.

### Regulatory capture of GLS genes by *MYB115* and *MYB118* goes beyond the new pathway

**Figure 10:**
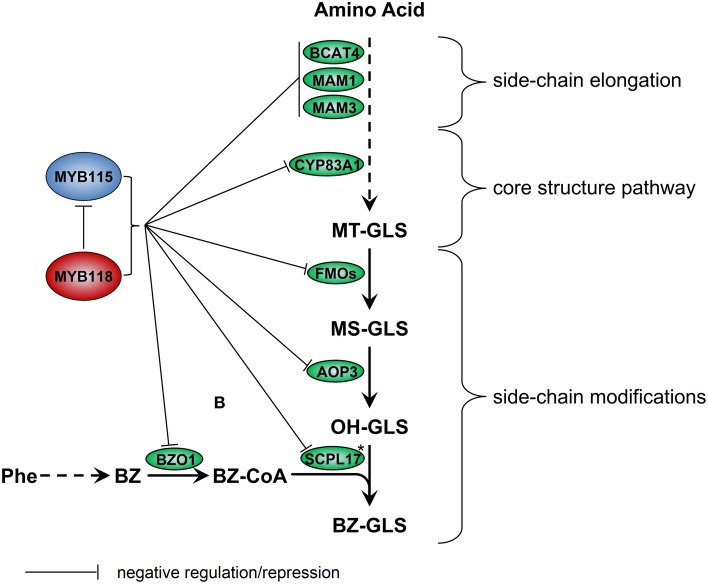
**Working model for regulation of GLS biosynthesis by *MYB115* and *MYB118* in *A. thaliana.*** For simplicity, only genes discussed in the text have been included. Enzymes are shown in circles. Asterisks partially characterized enzyme. Arrows show the combination of genetic and yeast one-hybrid evidence of how these MYBs influence the glucosinolate pathway transcription via an as to be yet identified molecular mechanism.

We identified the *MYB115* and *MYB118* TFs based on co-expression analysis with BZ-GLS related genes (*AOP3*, *BZO1*, and *SCPL17*) but these co-expression networks had no other GLS genes within them. However, using the yeast one-hybrid assays, it could be demonstrated that MYB115 and MYB118 interact more broadly with more genes of the aliphatic GLS biosynthetic pathway, including also *BCAT4*, *MAM1, MAM3, and CYP83A1* (Figure 2). Thus, this list of potential target genes encompasses genes involved in all three steps of aliphatic GLS biosynthesis, side-chain elongation (*BCAT4*, *MAM1*, and *MAM3*), core structure pathway (*CYP83A1*) and side-chain modification genes (*BZO1*, *SCPL17*, and *AOP3*). This was however not universal as there was no observed interaction with *BCAT3*. In agreement with this broader interaction capacity, the seed GLS profiles of the knockouts had alterations in GLS phenotypes not associated with the production of BZ-GLS (Figure 3; Table S2). The combination of yeast one-hybrid analysis with seed GLS profiles of the knockouts suggests that *MYB115* and *MYB118* have captured the capacity to regulate genes in all steps of the GLS pathway including the new BZ-GLS genes (Figure 10).

### *MYB115* and *MYB118* interact genetically

Our qRT-PCR work showed that in single mutant *myb118*, the transcript level of *AOP3* and *BZO1* increased in comparison with the wild type suggesting that MYB118 negatively regulate BZ-GLS related genes. In contrast, the single myb115 mutant had no altered expression of the *AOP3* and *BZO1* genes (Figure 2C). However, *AOP3* and *BZO1* transcript levels of double *myb115myb118* mutants were significantly higher than all single mutants and wild type indicating that *MYB115* had a genetic repressive activity on these genes that was redundantly in the presence of a functional *MYB118*. In agreement, the seeds GLS profiles of double mutant *myb115myb118* showed that *MYB115* and *MYB118* show a similar epistatic interaction in regulating the biosynthesis of most of GLSs. The combination of transcriptional analysis and seed GLS profiles of the knockouts suggests that there is an epistatic interaction between the two loci potentially via a regulatory interaction (Figure 10). This is not unprecedented within TFs controlling GLS genes. Work comparing single and double mutants of the *MYB28* and *MYB29* paralogs showed that they have a synergistic interaction potentially via the ability to regulate each other's gene expression (Sønderby et al., 2007, 2010a; Beekwilder et al., 2008; Gigolashvili et al., 2008). Further work is required to elucidate the molecular basis of the interaction between *MYB115* and *MYB118*.

### *MYB115* and *MYB118* are thought to be seed specific, but GLS in other tissues were also affected

Previous studies on *MYB118* and *MYB115* had suggested that these genes were largely functioning within the developing seed (Wang et al., 2009; Zhang et al., 2009; Barthole et al., 2014). This agreed with the observed expression pattern. Interesting, we could show that there was an effect of the *myb115* and *myb118* mutants on the accumulation of foliar GLSs (Figure 4; Table S2). This indicates that these genes have the capacity to influence the accumulation of GLSs in adult leaves before bolting. This however does not require these TFs to be directly GLS accumulation with in the leaves. One possibility is that because GLS can be transported in a bidirectional fashion within intact plants, there may be a unknown cell type in which they are expressed and affect global GLS patterning (Nour-Eldin et al., 2012; Madsen et al., 2014). For example, *MYB115* and *MYB118* do show some root expression under certain conditions (Gifford et al., 2008; Wang et al., 2009). Another more intriguing possibility is that by altering the accumulation of GLS in the developing seed, *MYB115* and *MYB118* alter downstream GLS regulatory decisions even when they are not present, effectively a maternal effect. Altering GLS accumulation can influence broad regulatory patterns suggesting that this is a possibility (Kerwin et al., 2011). Significant future work is required to test between these two potential indirect models of how *MYB115*/*MYB118* affects foliar GLS accumulation or if the residual expression of these genes in the leaf has direct consequences on the GLS accumulation in this tissue.

## Conclusion

Using a comprehensive strategy of co-expression analysis, we found that two conserved TFs, MYB115, and MYB118, regulate the genes in the newly evolved BZ-GLS pathway within *A. thaliana*. We validated that these two TFs play important roles in transcriptional regulation of aliphatic GLS biosynthetic pathway, which are negative regulators to be identified in GLS biosynthesis. Our study suggests that newly evolved metabolites can be regulated by conserved TFs; this has led to an improved model for newly evolved genes regulation. Future experiments will be required to understand how the promoters in this pathway evolved to allow coordinated expression within the developing seed via the use of conserved TFs.

## Materials and methods

### Plant materials and growth conditions

All plants were grown in controlled-environment chambers with 16 h light, 100 mE light intensity, 21°C/18°C day/night temperature, and 60% relative humidity. Wild type Col-0 controls were included in each flat to minimize any spatial aspects of the growth chamber. Before sowing, all seeds were stratified for 4 days in the dark at 4°C to break seed dormancy.

### Co-expression analysis

We performed a genome-wide co-expression analysis on BZ-GLS-related genes using ATTED-II (Obayashi et al., 2014). All these three genes were typed together (*AOP3*, AT4G03050; *BZO1*, AT1G65880, and *SCPL17*, AT3G12203) into CoExSearch box as query genes (http://atted.jp/top_search.shtml#CoExSearch). Under such a query, we got a list of the top 300 co-expressed genes for all the three genes. There are three MYB TFs among them, MYB58 (Rank#102, average correlation coefficient to query loci#0.22), MYB118 (Rank#198, average correlation coefficient to query loci#0.18) and MYB56 (Rank#291, average correlation coefficient to query loci#0.13).

### T-DNA insertion mutants and double mutants

T-DNA insertion lines were obtained from the Arabidopsis Biological Resource Center stock center (*myb115*, SALK_044168; *myb118*, SALK_111812; *aop3*, SALK_001655; *bzo1*, SALK_094196). Primers for genotyping are listed in Table S7. To construct the double mutant's *myb115myb118*, *myb115aop3*, *myb118zop3*, *myb115bzo1*, and *myb118bzo1*, the respective homozygous single knockouts were crossed with each other and F2 segregating progenies were genotyped to select homozygous mutations, and the F3 seeds were validated and used for the GLS phenotyping.

### Construction of complementation vectors and plant transformation

For the genetic complementation study, the T-DNA insertion mutants *myb115* and *myb118* were complemented with genomic fragments spanning the genomic region of *MYB115* (3998 bp) and *MYB118* (4692 bp), respectively, which were amplified with the Phusion High-Fidelity DNA Polymerase (Biolabs) by PCR using Col-0 genomic DNA as templates. (primers for cloning are listed in Table S7). This genomic fragment was introduced in vector pKGW-RR containing a red fluorescent protein (DsRed) (Varma Penmetsa et al., 2008) by TOPO and the Gateway system. The resulting binary vectors were electroporated into *Agrobacterium tumefaciens* GV3101 strain and used for agroinfiltration of flower buds of Arabidopsis (Bechtold et al., 1993). The homozygous seeds were screened by DSred fluorescence under the fluorescence microscope using dsRED-specific filter and further confirmed by PCR-based genotyping.

### Yeast one-hybrid

The yeast one-hybrid assay was performed according to the manual of Matchmaker Gold Yeast One-Hybrid Library Screening System (Clontech). Briefly, the open reading frames of *MYB115* (1080 bp) and *MYB118* (1314 bp) were cloned into the pGADT7 from Col-0 cDNA of leaf material by Phusion High-Fidelity DNA Polymerase (Biolabs), as prey vectors. Cloning primers are listed in Table S7. For the promoter cloning, about 2000 bp genomic fragments of each promoter upstream of the translational start codon were fused to pAbAi as the bait vectors. All the bait vectors including p53-AbAi (positive control) were firstly transformed into the Y1H Gold yeast strain. After transformants were selected on SD/-Ura plates by PCR, the minimal inhibitory concentration of aureobasidin A (AbA; Clontech) was determined for the bait strains. All the prey vectors including a mutant W-box-AbAi (negative control) were introduced into the respective Y1H Gold strain. The co-transformed yeast cells were cultured on SD/-Leu plates with and without AbA and incubated at 30°C until colonies in the positive control (p53) were visible.

### GLS extraction and analysis

For analysis of GLS content in all the genotypes, we planted four independent plants per genotype in a randomized design using 16 h long day length conditions. For leaf GLS analysis, we harvested one leaf from the first fully mature leaf pair of 4 week old plants. Under these conditions, the plants flower at about 7 weeks of age and as such, the plants were in mid-growth stage. This leaf was removed and stored in 90% (v/v) methanol to inhibit enzymatic breakdown of chemical compounds and begin the extraction. Five flowers per plant were collected 6 weeks after sowing and combined to make a single biological sample per plant. The plants were then allowed to continue to develop and produce seeds. For seeds samples, we collected mature seeds from each plant individually and extracted GLSs from 40 seeds. For the dissecting flower samples, sepal, petal, stamen and stigma were dissected, and 5 organs from each genotype are used for extraction. GLSs from all tissues were extracted and analyzed by HPLC according to previously described methods (Kliebenstein et al., 2001b). The entire experiment was replicated twice and the data combined for statistical analysis.

GLS contents were analyzed via ANOVA utilizing a general linear model within R software (x64 3.1.2) (R Development Core Team, 2014). For single mutants, each of them was tested for altered GLS content in an individual ANOVA against the wild-type Col-0. For the complementation tests, three independent transgenic lines of *myb115* and *myb118* were obtained and a nested model was used wherein the independent transgene lines were nested within the higher genotype term. In all models, the separate independent experiments were combined and an experiment term was included in the model to test for effects.

### Gene expression analysis

Developing seeds tissues were sampled at 6 and 7 days after pollination. For the expression study, nine independent biological samples spread across two experiments were utilized Total RNA was extracted with TRIzol Reagent according to the manufacturer's instructions (Life Technologies). The extracts were treated with 2 units of Rnase-free Dnase I (New England Biolabs) to eliminate the residual genomic DNA present in the preparation and eluted with 50 μl of RNase-free Water. For reverse transcription, first-strand cDNA was synthesized from 2 μg of total RNA using the Invitrogen ThermoScript RT-PCR system (Life Technologies). qRT-PCRs was performed with three technical replicates on a Bio-Rad CFX96 Real-Time system (Bio-Rad) and DBI Bioscience Bestar-Real Time PCR Master Mix kit, following the manufacturer's instructions (DBI Bioscience). Primers for qRT-PCR are listed in Table S7. The data were analyzed with LINREG, as described by Ramakers et al. (2003). The experiment was repeated using at least three independent biological replicates.

### Accession numbers

Sequence data from this article can be found in the GenBank/EMBL data libraries under the following accession numbers: *AOP3*, At4g03050; *BCAT3*, At3g49680; *BCAT4*, At3g19710; *BZO1*, At1g65880; *CYP83A1*, At4g13770; *MAM1*, At5g23010; *MAM3*, At5g23020; *MYB115*, At5g40360; *MYB118*, At3g27785; *SCPL17*, At3g12203; and *UBC21*, At5g25760.

### Conflict of interest statement

The authors declare that the research was conducted in the absence of any commercial or financial relationships that could be construed as a potential conflict of interest.

## Supplementary material

Figure S1 **Molecular characterization of *myb115*, *myb118*, *aop3*, and *bzo1* mutants**. Structure of the *MYB115* **(A)**, *MYB118* **(B)**, *AOP3* **(C)**, and *BZO1* **(D)** genes showing the position of T-DNA insertions in *myb115*, *myb118*, *aop3* and *bzo1* mutants are presented. Accumulation of *MYB115*, *MYB118*, *AOP3*, and *BZO1* mRNA in Col-0 and corresponding mutant backgrounds was measured by RT-PCR on developing seeds of 6 and 7 days after pollination. Ubiquitin-conjugating enzyme 21 (*UBC21*) gene expression was used as a constitutive control. Primers used for this study are indicated as arrows (see primers in Table S7).

Figure S2 **Expression pattern of *FMO*_***GS−OX2***_. (A)** The coexpression network analysis of *BZO1, SCPL17*, *MYB118*, and *FMO_GS−OX2_* using the ATTED-II database (Obayashi et al., 2014). The transcription factor and genes are shown in circles. **(B)** eFP display (Winter et al., 2007) of transcript accumulation patterns of *FMO_GS−OX2_*across a variety of *Arabidopsis* organs. In all cases, Red indicates higher levels of transcript accumulation and yellow indicates a lower level of transcript accumulation.

Figure S3 **GLS contents in leaves of *myb115*, *myb118*, and *myb115myb118* knockout mutants**. The data are combined from three independent experiments and analyzed via ANOVA. Asterisks are placed above a genotype to show if a main effect or interaction term is significant (*P* < 0.05). An asterisk above the *myb115* or *myb118* shows that there is a main effect of that gene within the ANOVA while an asterisk above *myb115myb118* shows that there was a statistically significant double mutant interaction indicating epistasis between *myb115* and *myb118*. **(A)** GLS content of 4-week-old rosette leaves. (Means and SE, *n* = 28). The data are combined from three independent experiments and analyzed via ANOVA. The significant differences is shown as * (*P* < 0.05) on the bar. **(B)** Statistical analysis of biosynthetic ratios of GLS data in **(A)**.
