# Supplementary figures and images for: The conserved transcription factors, MYB115 and MYB118, control expression of the newly evolved benzoyloxy glucosinolate pathway in Arabidopsis thaliana
Source: Front Plant Sci. 2015 May 13;6:343. doi: 10.3389/fpls.2015.00343 (PMC4429563; doi:10.3389/fpls.2015.00343)

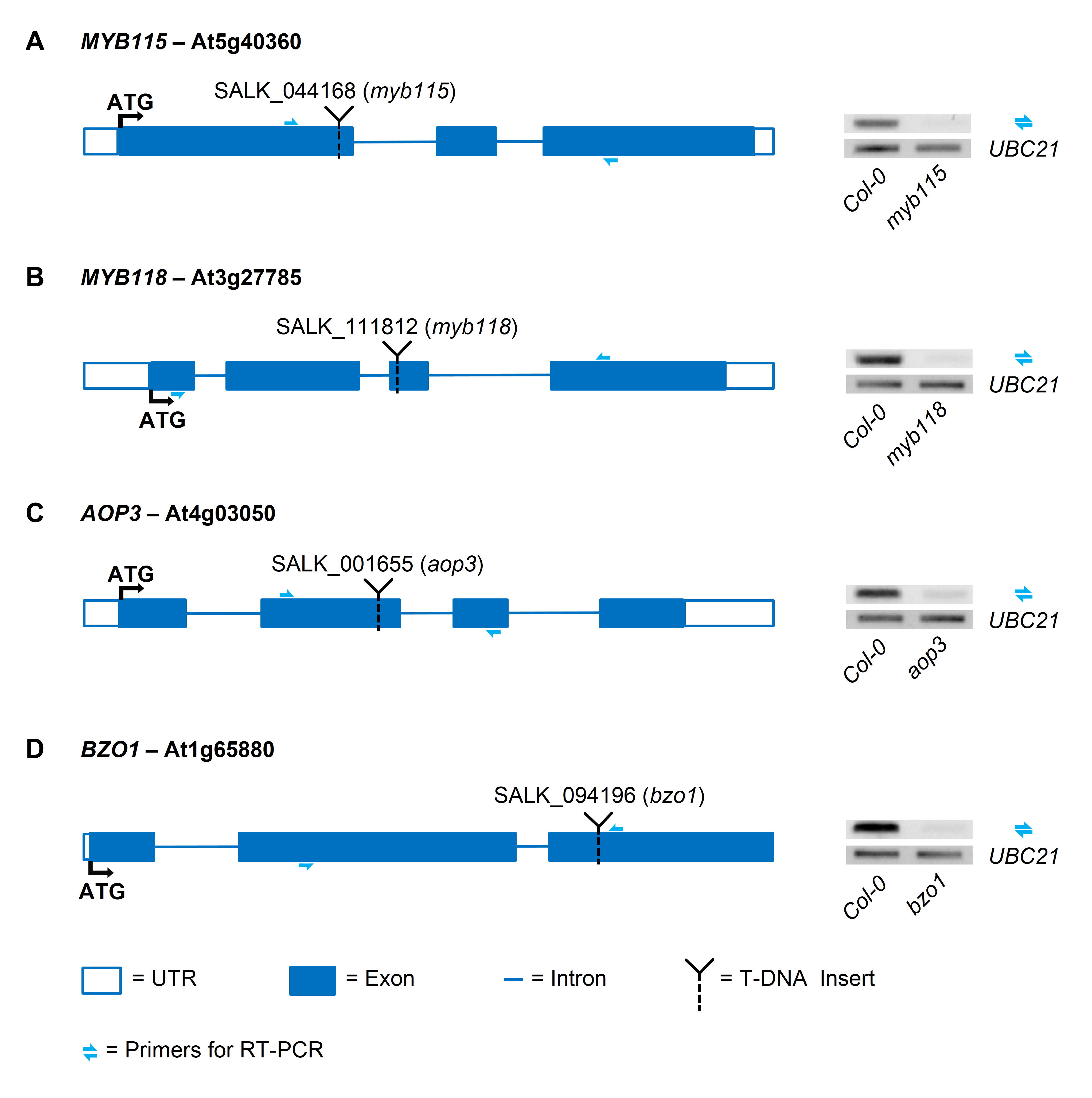

Supplement: Figure S1 — Molecular characterization of myb115, myb118, aop3, and bzo1 mutants. Structure of the MYB115 (A), MYB118 (B), AOP3 (C), and BZO1 (D) genes showing the position of T-DNA insertions in myb115, myb118, aop3 and bzo1 mutants are presented. Accumulation of MYB115, MYB118, AOP3, and BZO1 mRNA in Col-0 and corresponding mutant backgrounds was measured by RT-PCR on developing seeds of 6 and 7 days after pollination. [file Image1.TIF]

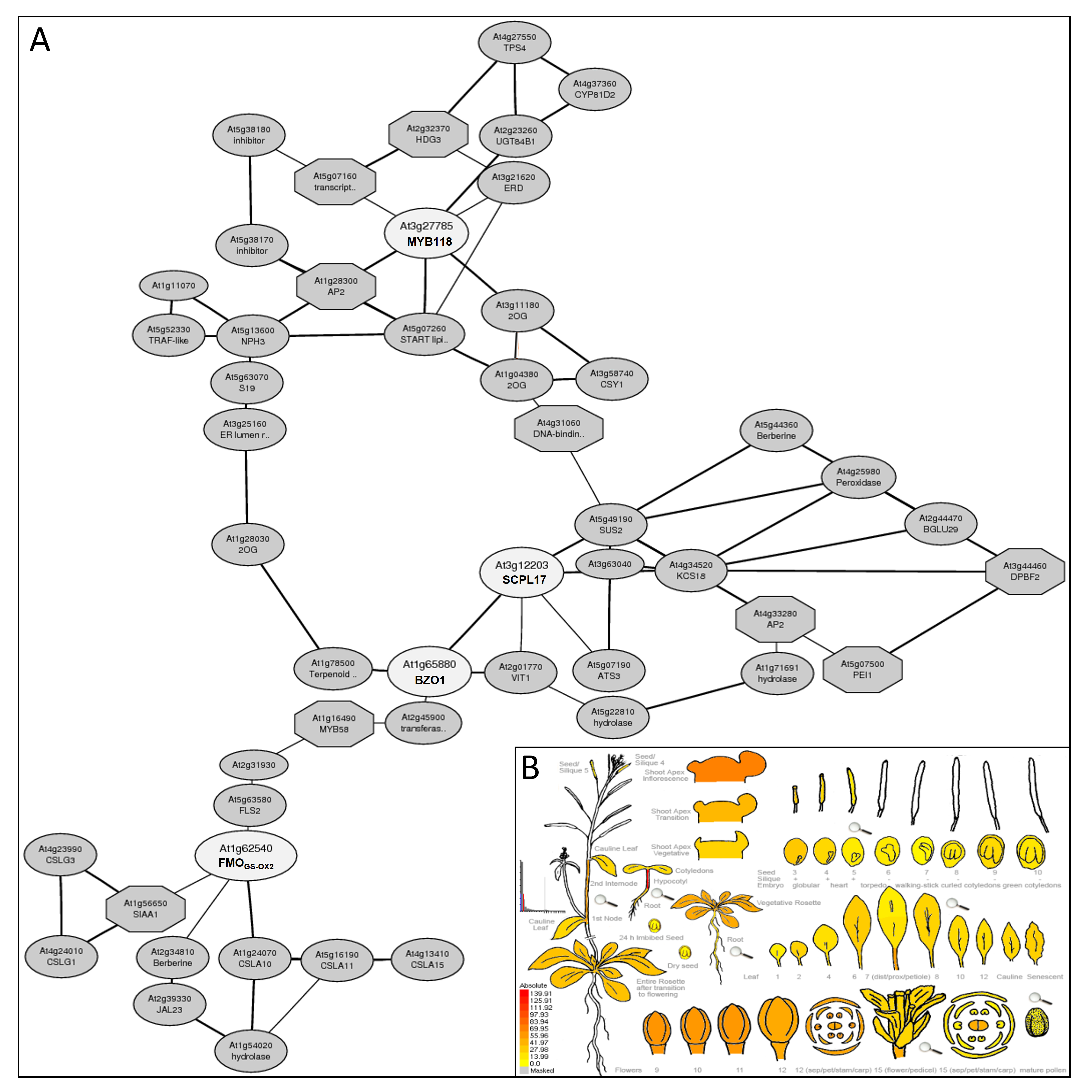

Supplement: Figure S2 — Expression pattern of FMOGS−OX2. (A) The coexpression network analysis of BZO1, SCPL17, MYB118, and FMOGS−OX2 using the ATTED-II database (Obayashi et al., 2014). The transcription factor and genes are shown in circles. (B) eFP display (Winter et al., 2007) of transcript accumulation patterns of FMOGS−OX2across a variety of Arabidopsis organs. In all cases, Red indicates higher levels of transcript accumulation and yellow indicates a lower level of transcript accumulation. [file Image2.TIF]

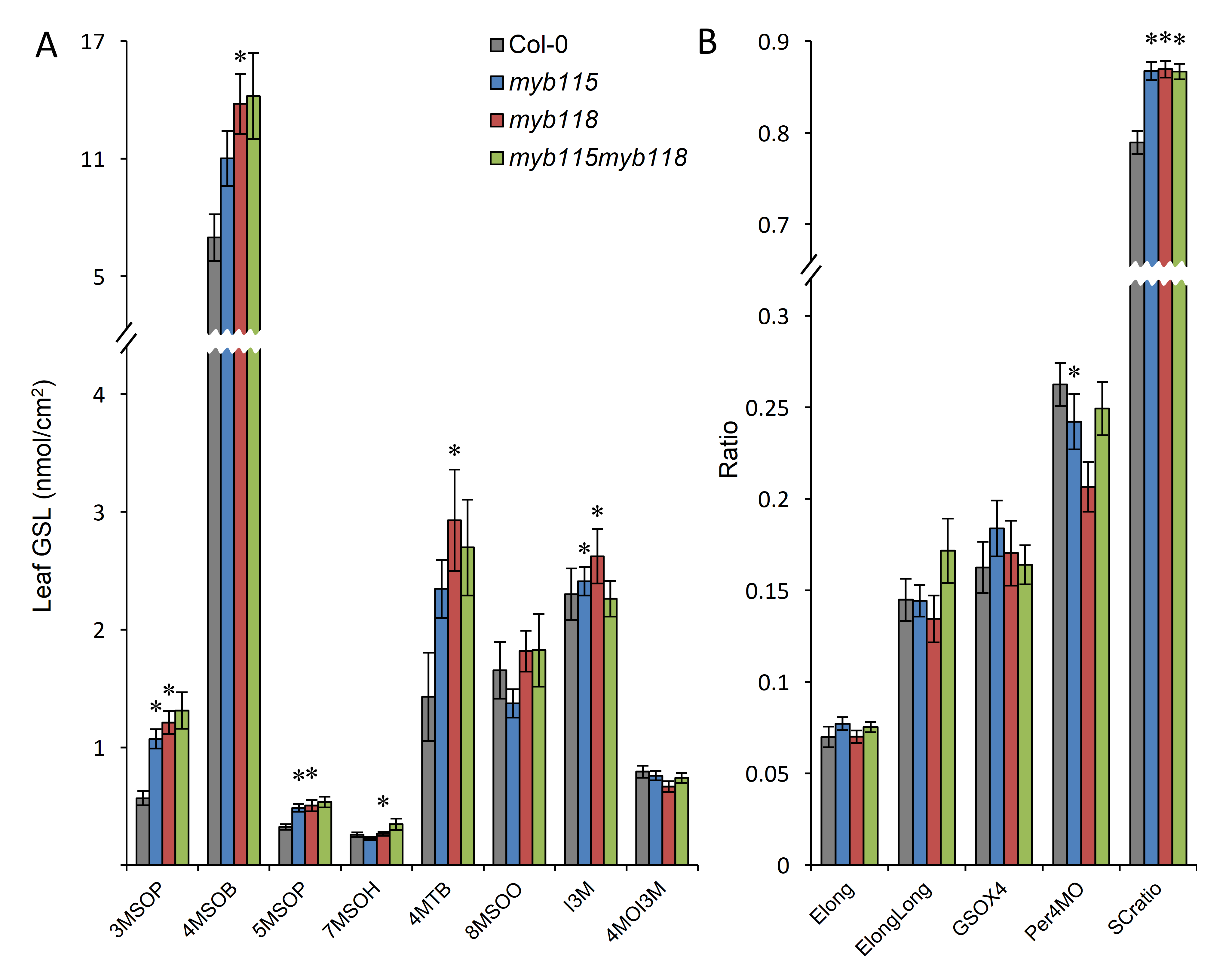

Supplement: Figure S3 — GLS contents in leaves of myb115, myb118, and myb115myb118 knockout mutants. The data are combined from three independent experiments and analyzed via ANOVA. Asterisks are placed above a genotype to show if a main effect or interaction term is significant (P < 0.05). An asterisk above the myb115 or myb118 shows that there is a main effect of that gene within the ANOVA while an asterisk above myb115myb118 shows that there was a statistically significant double mutant interaction indicating epistasis between myb115 and myb118. (A) GLS content of 4-week-old rosette leaves. (Means and SE, n = 28). The data are combined from three independent experiments and analyzed via ANOVA. The significant differences is shown as * (P < 0.05) on the bar. (B) Statistical analysis of biosynthetic ratios of GLS data in (A). [file Image3.TIF]
